# The effect of the stay active advice on physical activity and on the course of acute severe low back pain

**DOI:** 10.1186/s13102-015-0013-x

**Published:** 2015-08-27

**Authors:** Patricia Olaya-Contreras, Jorma Styf, Daniel Arvidsson, Karin Frennered, Tommy Hansson

**Affiliations:** 1 Department of Orthopedics, Institute of Clinical Sciences at the Sahlgrenska Academy, University of Gothenburg, Gothenburg, Sweden; 2 Department of Postgraduate Studies, Faculty of Nursing, University of Antioquia, Calle 70 No 52-21, Apartado Aereo, 1226 Medellín, Antioquia Colombia; 3 Unit for Health Promotion Research, University of Southern Denmark, Esbjerg, Denmark; 4 Unit of Clinical Physiology and Nuclear Medicine, Department of Translational Medicine, Lund University, Malmö, Sweden; 5 RICH/EXE, Institute of Sports Science and Clinical Biomechanics, University of Southern Denmark, Odense, Denmark

## Abstract

**Background:**

Disability due to acute low back pain (ALBP) runs parallel with distress and physical inactivity. If low back pain persists, this may lead to long-term sick leave and chronic back pain. This prospective randomized study evaluated the effect on physical activity and on the course of ALBP of two different treatment advices provided in routine care.

**Methods:**

Ninety-nine patients with acute severe LBP examined within 48 h after pain onset were randomized to the treatment advices “Stay active in spite of pain” (stay active group) or “Adjust activity to the pain” (adjust activity group). Pedometer step count and pain intensity (Numeric Rating Scale, NRS, 0–10) were followed daily during seven days. Linear mixed modeling were employed for statistical analyses.

**Results:**

The step count change trajectory showed a curvilinear shape with a steep initial increase reaching a plateau after day 3 in both groups, followed by an additional increase to day 7 in the stay active group only. At day 1, the step count was 4560 in the stay active group compared to 4317 in adjust activity group (*p* = 0.76). Although there were no statistical differences between the two groups in the parameters describing the change trajectory for step count, the increase in step count was larger in the stay active group. At day 7 the step count was 9865 in the stay active group compared to 6609 in the adjust activity group (*p* = 0.008). The pain intensity (NRS) trajectory was similar in the two groups. Between day 1 and day 7 it decreased linearly from 5.0 to 2.8 in the stay active group (*p* < 0.001), and from 4.8 to 2.3 in the adjust activity group (*p* < 0.001).

**Conclusions:**

Patients with acute severe LBP advised to stay active in spite of the pain exhibited a considerable more active behavior compared to patients adjusting their activity to pain. This result confirms compliance to the treatment advice as well as the utility of the stay active advice to promote additional physical activity for more health benefits in patients with ALBP. There was minimal effect of the treatment advice on the course of ALBP.

**Trial registration:**

ClinicalTrials.gov (NCT02517762).

## Background

The prevalence of low back pain (LBP) is around 10 % and it causes more disability than any other condition [1]. The highest prevalence can be found in Western Europe, with almost 16 % of the males and 15 % of the females affected [1]. Acute low back pain (ALBP) defined as an episode of LBP persisting for less than six weeks [2], is commonly encountered in primary care practice. Nevertheless, often the specific cause cannot be identified in spite of a variety of diagnostic methods in general practice. A specific diagnosis can only be reached in around 10–20 % of all patients with LBP. Even though ALBP has good prognosis with normalization of its symptoms usually within few days, as many as 30 % of people with episode of nonspecific LBP do not recover within 1 year [3, 4]. Additionally, the risk for recurrence and development into chronic LBP is between 2 % and 56 % [5–7]. About half of the adult population will suffer from LBP during a 12-month period [8]. In Sweden, the high prevalence of spine problems is a major source of disability and treatment for this necessitates high levels of health care expenditure [9]. Therefore, regimens that accelerate recovery of ALBP would be of profound importance for optimizing clinical practice, which could prevent chronicity of pain and reduce a big amount of disability due to LBP.

There is substantial evidence that physical activity has beneficial effects on most musculoskeletal conditions, including LBP [10]. For that reason, advising the patient to stay active is a crucial part of the recommended treatment of ALBP [2, 11]. However, current evidence in favor for the stay active advise in patients with ALBP is limited, with small or no benefits in pain relief, functional improvement or sick leave compared to rest in bed [12]. Effects in favor for rest in bed has also been reported [12]. In an observational study, lower risk of ALBP and lower rate of recurrence were found among patients advised to stay active compared to patients advised to rest [13]. In these studies, the stay active advice was implemented several days after onset ALBP, and an important part of its effect may therefore have been lost. In addition, as the symptoms of ALBP have a course of days up to a week, late assessment would probably lead to lost treatment opportunities to support patients to stay active and to prevent negative pain behaviors/pain avoidance. Previous studies investigated the effect of the stay active advice after several months [12]. However, to best of our knowledge, no study has actually investigated neither the early implementation of the stay active advice after a severe ALBP, nor followed up its effect on pain or compliance to treatment advice using an objective measure of physical activity, prospectively.

Compliance to the stay active advice could be an important factor influencing the magnitude of the effect on ALBP, but little has been reported. Malmivaara et al. found less hours of bed rest and more hours doing back exercises as measures of compliance in patients with ALBP receiving a stay active advice compared to patients advised to rest in bed. In the referred study, compliance was assessed by means of a questionnaire [14], thus, these questions were not direct measures of whether the patients stayed active and maintained their normal activity levels. Further, questionnaires are prone to recall bias and may exaggerate any intervention effect [15, 16]. It is likely that bed rest is a rather obsolete advice for patients with ALBP today. Currently, stay active or adjust your activity according to the pain are probably the most common clinical advices. However, the definition and implementation of the stay active advice may vary between clinics and investigators. A more cautious attitude among general practitioners may influence the beliefs of the patient and compliance to intentioned treatment [17–20].

Moreover, fear avoidance beliefs have been shown to influence the prognosis of ALBP [21–23]. According to previous research, pain avoidance belief in general practitioners is associated with prescribing sick leave during painful periods of ALBP [24, 25]. Further, management of first time ALBP varies, reflecting uncertainty about the optimal approach [25, 26]. Therefore, there is a need for implementation of early treatments strategies relying on evidence-based knowledge to treat acute problems and lower the risk for recurrence and chronicity of LBP.

The stay active advice may not only be a treatment to improve recovery from ALBP, but also an opportunity to promote physical activity for other health benefits, such as improved cardio-metabolic function, blood pressure, and reduced body fatness [27, 28]. An individual that accumulates at least 10000 steps daily could be defined as being at a health-enhancing level of physical activity [29]. However, a low proportion of the general population actually meets the recommended level of physical activity. Among person with low level of physical activity it has been observed increased risk for LBP, recurrence and disability due to LBP [30].

In the present study, the two treatment advices “Stay as active as possible in spite of the back pain” or “Adjust activity to pain” were implemented early after onset of acute severe LBP. The aims were to evaluate their effect on objectively measured physical activity and on the course of ALBP.

## Methods

### Design

A prospective randomized study was conducted at the Department of Orthopaedics, Sahlgrenska University Hospital Gothenburg, Sweden, to evaluate the effect on physical activity and on the course of acute severe LBP of two different treatment advices provided in routine care. All patients were followed for seven days from maximum 48 h after the onset of the ALBP. The Regional Ethical Review Board at the University of Gothenburg approved the study protocol. Trial registration: ClinicalTrials.gov (NCT02517762).

### Patients and procedures

Participants in the study were recruited consecutively among employees from a large local manufacturing company representing several different factories. All employees had been informed to immediately contact the company physiotherapist or the nurse coordinating the study in case of acute severe pain in the lower back. Eligible participants were subjects between 18 and 65 years of age, with acute severe LPB, with duration from onset less than or equal to 48 h, with or without radiating leg pain, with or without neurological signs, and the pain had to exceed 50 mm on the Visual Analog Scale (VAS). Patients were requested to fill out and return a seven-day diary and those who did so were included. Excluded were those who had been on sick leave because of LBP in the last month or because of pain in the spine. Employees determined eligible were enrolled in the study after giving informed consent, and were immediately referred to an academic orthopedic department for further examinations. Enrolment took place from March 2005 until December 2008.

At the hospital the patients underwent an X-ray examination of the lumbar spine (frontal and lateral projections and a spot view of the lumbosacral spine) followed by a magnetic resonance imaging (MRI) examination including T1 and T2 weighted and short time inversion recovery (STIR) sequences. They also underwent an extensive physical examination performed in a standardized way by one of three orthopedic spine specialists. The physician explained for the patient the imaging findings as well as the results of the physical examination. The patients were also asked to complete a battery of questionnaires [31] covering history of ALBP, lifestyle characteristics, work place factors, and initial intensity of pain on Visual Analog Scale (VAS), and location of pain on pain drawing. Additional psychosocial factors and psychological variables were asked.

**Fig. 1:**
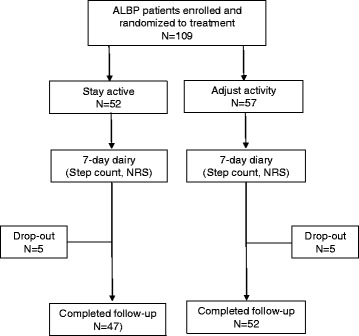
The patient flow chart

Thereafter, each patient was randomly allocated to one of the two treatment advices, using a random table. A sealed envelope with the treatment assignment was distributed to the physician, who instructed the patient about the content and practical aspects of the actual treatment advice according to protocol (see Treatments below). To obtain as similar information as possible the three physicians coordinated the content of the two treatment advices prior to the study. This coordination was repeated at several occasions during the study period to keep the instruction as constant as possible. The coordinating nurse gave the patient instructions regarding the 7-day diary (see 7-day diary below). The patient was instructed to return the completed diary as soon as possible after the follow-up period. One month after entering the study, each patient had a follow-up appointment with a physiotherapist at the company health center to check the patient’s status. Throughout the entire study, the coordinating nurse acted as a study monitor, guiding each patient through the study and providing standardized information. We enrolled and allocated 109 employees to treatment (Fig. 1).

### Treatment advices

The patients were advised either to stay as physically active as possible in spite of the LBP (stay active, SA), or to adjust the activity according to the pain (adjust activity, AA). Patients with the AA advice were instructed to avoid activities, movements, or positions that caused or worsened the pain. Of the 109 randomized patients, 52 patients (47.5 %) were allocated the SA advice and 57 patients (52.5 %) to the AA advice.

### Medication

All the patients who wanted help with pain relief were prescribed either paracetamol and/or NSAID. The number of prescriptions in the two groups was similar but the use of the drugs was not checked.

### 7-day diary

After the clinical examinations and the allocation to the treatment, each patient received a diary to record daily step count (pedometer), pain intensity (NRS), pain location and pain-related disability (DRI) during the following 7 days. In addition, they described in the diary all kind of physical activities performed under the 7-day follow-up period.

### Physical activity

Step count was used both as a measure of daily physical activity, and as an indicator of compliance to treatment advice over the 7-day follow-up. Each patient received a digital pedometer (LS 2000, Kalmar, Sweden) and was instructed to wear it during all waken hours and to record the daily step count in the diary. This type of pedometer has been validated in previous studies for estimating the total daily number of steps [32, 33]. The daily step count was used to categorize patients according to step count cut-offs for activity levels defined from healthy adults: sedentary <5000, low active 5000–7499, somewhat active 7500–9999, and active ≥10000 [34]. The last category has also been used as the recommended level of step count to promote health. The patients were also asked to report in the diary any other kind of special physical activities that they participated in at work or during leisure (e.g. sporting events, etc.), during the follow up.

### Pain intensity

The Numerical Graphic Rating Scale (NRS, 0–10) is a box scale consisting of 11 numbers from 0 (no pain) to 10 (pain as bad as it could be). The patients were asked to place an “X” at the number that represented their pain. The NRS is easy to administer and there is good evidence for its construct validity [35]. The VAS was used to self-assess the patient’s pain intensity at the initial clinical examination, and was rated on a 100 mm scale, ranging from 0 (no pain) to 100 (worst possible pain) [35]. In addition, using a full-body drawing in the dorsal and ventral views, the patient marked the location(s) of the pain [35].

### Absenteeism

Information regarding work absenteeism and sick leave due to the current back problems was collected from the company records up to one month after the onset of the ALBP episode.

### Statistical analyses

Due to the lack of previous information on step count from patients with ALBP, a power calculation was performed as follows. Based on an estimated mean difference in daily step count between the 2 groups of 1000 steps (SD = 2000/day) reported by healthy subjects [36], to achieve a statistical power of 0.80 with a significance level of 0.05, it was estimated that 120 patients would be required for this study [37]. Allowing for a dropout rate of up to 10 %, the target recruitment number was 66 patients in each group.

Group comparisons at baseline were performed using the Chi-squared test (gender, occupation, and type of activity before the LBP), the Mann–Whitney test (NRS, VAS), and the *t*-test (age and days of absenteeism). Linear mixed models (LMM) were used to estimate the shape of the step count and pain intensity (NRS) change trajectories over seven repeated measures (Day, 1–7), as they provide greater flexibility to repeated measures designs and their specific variance structures [38]. A third-order polynomial function provided the best fit to data for the change in step count over time, while a first-order polynomial function provided the best fit to data for pain intensity according to the Bayesian information criterion (BIC) for goodness-of-fit. Models developed included both fixed and random effects for intercepts, and fixed effects for all slope components (linear, quadratic and cubic terms). As we are limited in the number of random effects by the number of repeated measures, a random effect was included only for the linear slope component to describe inter-individual difference in change trajectory [38]. A second step was to include the fixed effect of treatment advice on intercepts and slope components. Maximum likelihood (ML) was used for the estimation of fixed and random effects. For the models developed, day 1 was used as intercept. By also defining the intercept at each of the other days (day 2–7), difference in step count or pain intensity could be statistically tested for each day over the entire follow-up. Statistical significance was set at *p* < 0.05. All statistical analyses were performed using SPSS 22 (IBM Coperation, NY, USA).

## Results

### Patients and clinical findings

One hundred-and-nine participants with acute (≤48 h) severe LBP (VAS > 50 mm) were enrolled in the study. The mean age for all the participants was 42.1 years (range 20–63). Seventy-two percent were men and 57 % percent were white-collar. Thirty-five percent of the patients claimed that their ALBP arose while working and 32 % reported that their back problems arose without any obvious external exertion. The diagnoses (ICD10 coding) were acute lumbago in 88 % (M545), acute lumbago with sciatica in 10 % (M544), and lumbar spinal stenosis in 2 % (M480). The majority of the patients (76 %) returned directly to work after the clinical examination, whereas 17 % were absent from work less than 5 days, and 7 % were absent from work between 6 to 8 days. The return to work rate was the same in the two treatment groups. There were no differences between the two groups for age, gender or sick leave due to the ALBP (*p* > 0.05). In addition, there were no differences between the groups regarding the reported cause of ALBP, occupation or initial pain intensity (VAS).

### Non-response analyses

Ninety-nine patients (91 %) completed and returned the diary with the information regarding step count, pain intensity (NRS) and pain-related disability (DRI). The average age was 37.3 years (range 27–53) for those not returning the diary, and 42.5 years (range 20–63) for those returning the completed diary (*p* > 0.05). There were no statistically significant differences between the responders and non-responders regarding gender or ethnicity (*p* > 0.05). Differences in the initial scores on DRI were found between the groups, where the responders scored higher (*p* < 0.05). For the responders included in the statistical analyses, 47 patients were assigned to the stay active group (SA) and 52 to the adjust activity group (AA) (Fig. 1). Of the 10 non-responders, 5 had been randomized to the SA group and 5 to the AA group.

### Physical activity change trajectory

**Fig. 2:**
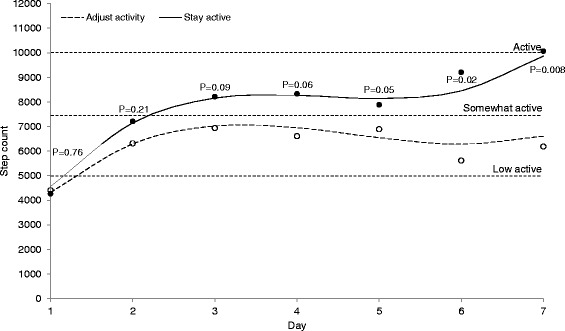
Modeled daily step count over the 7 days follow-up in patients with ALBP. Solid line represents patients advised to stay active (SA) and dashed line patients advised to adjust activity (AA) to the pain; horizontal lines are cut-offs for daily step count levels; p-values indicate statistical difference between groups of modeled values; circles indicate mean of observed step count (SA = filled, AA = open)

Figure 2 displays the modeled change trajectory of step count over time and is complemented by the results in Table 1. There was a steep initial linear increase (Table 1, Model 1, linear term, *p* < 0.001) that leveled off and reached a plateau after day 3 (quadratic term, *p* < 0.001). From day 6 there was an additional increase in step count (cubic term, *p* < 0.001). However, the change trajectory in step count was not similar in the two treatment groups. At the first follow-up day there was only a small difference of 243 steps between the groups (Model 2, intercept *p* = 0.76). Although there was no statistically significant effect of treatment advice on any of the three change trajectory terms (Model 2, linear *p* = 0.30, quadratic *p* = 0.42, cubic *p* = 0.34), the increase in step count was larger in the SA group compared to the AA group. At the plateau at day 3 the difference between the groups was 1133 steps (*p* = 0.09). Thereafter, the step count increased only in the SA group with statistically significant difference between groups reached at day 6 (*p* = 0.02, Fig. 2). At the last day of the follow-up period the estimated step count in the SA group was 9865 steps which approached the step count cut-off defined as being active, compared to 6609 steps in the AA group remaining in the low active step count category (*p* = 0.008). At the first day of the follow-up, 2 % in the SA group and 8 % in the AA group reached the recommended 10000 steps. At the last day, the corresponding proportions were 39 % in the SA group compared to 8 % in the AA group.

**Table 1:** Linear mixed models to estimate change in step count over seven days follow-up and the effect of treatment advice (SA = Stay active versus AA = Adjust activity) on this change

*N* = 99	Model 1: Change trajectory		Model 2: Effect of treatment advice	
Fixed effects in model	Estimate (SE)	*p*-value	Estimate (SE)	*p*-value
Day 1 (intercept)	4434 (405)	<0.001	4317 (558)	<0.001
+Stay active advice	-	-	243 (812)	0.76
Day (β, linear term)	3160 (398)	<0.001	2773 (545)	<0.001
+Stay active advice	-	-	824 (796)	0.30
Day^2^ (β, quadratic term)	−987 (161)	<0.001	−865 (220)	<0.001
+Stay active advice	-	-	−257 (321)	0.42
Day^3^ (β, cubic term)	94 (18)	<0.001	75 (24)	0.002
+Stay active advice	-	-	34 (36)	0.34

Fixed effects in models are presented with step count as outcome

Model 1 is the estimated change trajectory without the effect of treatment advice. Model 2 includes the effect of treatment advice, where the effect of the Adjust activity is presented first followed by the added effect of the Stay active advice

A third-order polynomial function was used with a linear term (Day) describing the initial increase (positive value), a quadratic term (Day^2^) describing the level-off of the initial increase (negative value) and a cubic term (Day^3^) for the additional final increase in step count over time

### Pain intensity change trajectory

**Fig. 3:**
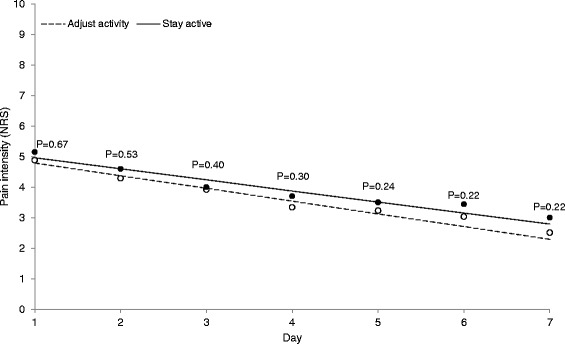
Modeled daily pain intensity (NRS) over the 7 days follow-up in patients with ALBP. Solid line represents patients advised to stay active (SA) and dashed line patients advised to adjust activity (AA) to the pain; p-values indicate statistical difference between groups of modeled values; circles indicate mean of observed pain intensity (SA = filled, AA = open)

Figure 3 and Table 2 display the modeled change trajectory of pain intensity (NRS) over time. The pain intensity decreased linearly over the follow-up period for all the patients in both groups (Table 2, Model 1, linear term *p* < 0.001). The SA group showed somewhat higher pain intensity and a somewhat slower decrease in pain intensity compared to the AA group, however, there was no statistically significant effect of treatment advice on the pain intensity change trajectory (Model 2, intercept *p* = 0.67, linear term *p* = 0.52). The estimated pain intensity decreased between day 1 and day 7 from 5.0 to 2.8 (*p* < 0.001) in the SA group, and from 4.8 to 2.3 (*p* < 0.001) in the AA group.

**Table 2:** Linear mixed models to estimate change in pain intensity (NRS) over seven days follow-up and the effect of treatment advice (SA = Stay active versus AA = Adjust activity) on this change

*N* = 99	Model 1: Change trajectory		Model 2: Effect of treatment advice	
Fixed effects in model	Estimate (SE)	*p*-value	Estimate (SE)	*p*-value
Day 1 (intercept)	4.9 (0.2)	<0.001	4.8 (0.3)	<0.001
+Stay active advice	-	-	0.2 (0.4)	0.67
Day (β, linear term)	−0.4 (0.04)	<0.001	−0.4 (0.06)	<0.001
+Stay active advice	-	-	0.05 (0.08)	0.52

Fixed effects in models are presented with pain intensity (NRS) as outcome

Model 1 is the estimated change trajectory without the effect of treatment advice. Model 2 includes the effect of treatment advice, where the effect of the Adjust activity is presented first followed by the added effect of the Stay active advice

A first-order polynomial function was used including the linear term (Day) describing a decrease in pain intensity over time (negative values)

## Discussion

The present study contributed with the follow-up of the effect of two treatment advices on objectively measured physical activity and on the course of ALBP from early after its onset. The patients advised to stay active (SA) in spite of LBP increased their activity more than the patients advised to adjust activity (AA) to the pain, which confirmed compliance with the advices. Due to this compliance and the early inclusion after the pain onset, it can be stated that the SA advice did not alter the course of ALBP. However, the SA advice promoted a pronounced increase in daily activity among these patients who reached the recommended level of 10000 steps for additional health benefits [34, 36, 39], which has important clinical and public health implications.

In a supportive clinical environment where general practitioners have a positive attitude to active rehabilitation in combination with early assessment and treatment, the fear of motion or avoidance of pain among patients advised to be physically active might be less pronounced [17–20]. Among patients receiving the SA advice, the large increase in step count with a large proportion of patients reaching the recommended level of step count should indicate that they overcame their fear of movement/activity related to pain, in line with previous research [21–23].

Previous research often involved ALBP patients from primary care settings recruited after three or more days of LBP duration [2, 14, 40, 41]. In the present study, the severe pain symptoms were alleviated within hours after presentation and the pain intensity decreased linearly over the follow-up period with the patients being cured or having at most a mild degree of pain at day 7. If the patients would have been examined later than within 48 h after the pain onset, many of them would not have been included in the study. Furthermore, the effect of the SA advice might be underestimated if started at a later stage of the course of ALBP. Our results shown that the SA advice and pedometer, as accessible methods, promoted a considerable larger increase in physical activity among patients in the SA group, even though they exhibited similar experience of pain as did the patients in the AA group. These findings have important clinical implications and thus, general practitioners should stimulate early activity and return to work among patients with acute lumbago.

One could argue that a patient whose back problem decays within a week is a minor clinical problem. However, even with the very short duration in the majority of patients with ALBP, there is a considerable risk for future recurrence and/or development into chronic LBP, as previously stated [5–7]. The combination of the SA advice and monitoring of step counts is an inexpensive treatment to maintain or even improve daily activity, instead of embracing a pain avoidance attitude, which commonly has been observed among general practitioners [17–20]. The evidence supports the beneficial effects of physical activity on most musculoskeletal conditions, including LBP [10]. Previous studies with a follow-up period of up to 12 weeks found favorable effects of advising the patient to stay active on pain intensity, functional status and sick leave compared to bed rest, although the effects were not consistent across studies [12]. The present study focused on the early, natural course of ALBP. A continuation would be to demonstrate effects of staying active on future recurrences of back pain, functional status, and sick leave among patients with acute severe LBP.

### Strengths and limitations

One strength in the herein study is the use of objective measure of physical activity, which has not been used in prior research to confirm compliance with the stay active advice in patients with acute severe LBP. Subjective methods tend to exaggerate intervention effects related to physical activity [16]. The use of pedometer for self-monitoring of behavior is an effective technique in itself to promote physical activity [36, 39], and as all the patients in the present study wore pedometers, the group differences found were likely attributed to the treatment advices. However, we cannot rule out the possibility of synergy between the SA advice and self-monitoring contributing to a larger increase in step count in the SA group. In the present study, the early, careful, and comprehensive examinations by experienced orthopedic spine specialists at a university clinic, which represent an optimal treatment condition for ALBP, could have influenced the compliance with the treatment advices. However, this bias was similar in both groups. Another strength is the inclusion of patients with ALBP from the very earliest hours after onset pain, i.e. referring severe pain, and comparing the treatment advices effect on pain and physical activity, which has not been performed previously. The AA advice might better reflect the advice provided in health care today, rather than the advice to stay in bed that has been used in previous research. The inclusion of patients in the study occurred over an extended period in order to try to reach the numbers determined in the power analysis (See Methods). In spite of the prolonged recruiting time, the study was forced to close before the optimal number of patients was obtained, due to economic and logistic reasons. Still, the effect of the SA advice versus the AA advice largely exceeded the 1000 steps difference considered prior in the power-calculations. The study is limited to the course of ALBP, not allowing conclusions either of long-term effects on pain, or recurrence/work absence due to chronic LBP.

## Conclusions

Treatment advice given in acute severe LBP is complied with. Patients advised to stay active showed a more active behavior compared to patients advised to adjust their activity to the pain. A large proportion in the SA group reached recommended level of 10000 steps per day defined for a population without pain. Thus, the stay active advice is appropriate for the early treatment of acute severe LBP and to promote additional physical activity for more health benefits among workers in risk for suffer from LBP.

### Practical applications

The present study demonstrates the opportunity within the health care setting to support return to habitual levels of physical activity and early return to work after onset of acute severe LBP, using inexpensive methods in form of the stay active advice and pedometers. It also demonstrates that even higher levels of physical activity can be promoted with these methods to prevent recurrence/chronicity of pain and for additional health benefits.

## Abbreviations

AA: Adjust activity
ALBP: Acute low back pain
LBP: Low back pain
NRS: Numeric Rating Scale
SA: Stay active
